# Mitophagy-associated genes *PINK1* and *PARK2* are independent prognostic markers of survival in papillary renal cell carcinoma and associated with aggressive tumor behavior

**DOI:** 10.1038/s41598-020-75258-4

**Published:** 2020-11-02

**Authors:** Adrian Georg Simon, Yuri Tolkach, Laura Kristin Esser, Jörg Ellinger, Christine Stöhr, Manuel Ritter, Sven Wach, Helge Taubert, Carsten Stephan, Arndt Hartmann, Glen Kristiansen, Vittorio Branchi, Marieta Ioana Toma

**Affiliations:** 1 Institute of Pathology, University Hospital Bonn, Venusberg-Campus 1, 53127 Bonn, Germany; 2 Department of Urology, University Hospital Bonn, Venusberg-Campus 1, 53127 Bonn, Germany; 3 Institute of Pathology, University Hospital Erlangen, Krankenhausstr. 8-10, 91054 Erlangen, Germany; 4 Department of Urology, University Hospital Erlangen, Krankenhausstr. 12, 91054 Erlangen, Germany; 5 Department of Urology, University Hospital Berlin-Charité, Charitéplatz 1, 10117 Berlin, Germany; 6 Department of General, Abdominal, Thoracic and Vascular Surgery, University Hospital Bonn, Venusberg-Campus 1, 53127 Bonn, Germany

**Keywords:** Cancer, Biomarkers, Urology

## Abstract

The aim of this study was to investigate the mitophagy-related genes *PINK1* and *PARK2* in papillary renal cell carcinoma and their association with prognosis. In silico data of *PINK1* and *PARK2* were analyzed in TCGA cohorts of papillary renal cell carcinoma comprising 290 tumors and 33 corresponding non-neoplastic renal tissues. Protein expression data from a cohort of 95 papillary renal cell carcinoma patients were analyzed and associated with clinical-pathological parameters including survival. *PINK1* and *PARK2* were significantly downregulated in papillary renal cell carcinoma at transcript and protein levels. Reduced transcript levels of *PINK1* and *PARK2* were negatively associated with overall survival (*p* < 0.05). At the protein level, PARK2 and PINK1 expression were positively correlated (correlation coefficient 0.286, *p* = 0.04) and reduced *PINK1* protein expression was prognostic for shorter survival. Lower PINK1 protein levels were found in tumors with metastases at presentation and in tumors of higher pT-stages. The multivariate analysis revealed mRNA expression of *PINK1* and *PARK2* as well as PINK1 protein expression as independent prognostic factors for shorter overall survival. The downregulation of PINK1 is a strong predictor of poor survival in papillary renal cell carcinoma. Immunohistochemical PINK1 expression in resected pRCC should be considered as an additional prognostic marker for routine practice.

## Introduction

Papillary renal cell carcinoma (pRCC) is the second most common subtype of renal cell carcinoma (RCC) after clear cell RCC (ccRCC). It accounts for 10–15% of all renal cancer cases^1^. Clinical manifestations of pRCC are heterogeneous. While some patients are diagnosed with multiple, bilateral yet indolent lesions, others are burdened with aggressive, highly invasive and metastatic tumors^2^. According to their histology, pRCC specimens are sub-classified into type 1 and 2. This sub-classification correlates to specific molecular profiles^3^. Type 1 pRCC (basophilic pRCC) often carries MET gene alterations, whereas type 2 (eosinophilic) tumors are associated with *CDKN2A* silencing, *SETD2* mutations, and an increased expression of the NRF2-antioxidant response element (ARE) pathway^4^.

- Although pRCC is the second most frequent RCC, predictive molecular biomarkers are still lacking. Cancer-specific metabolism products, cell cycle or cell surface proteins involved in cell–cell interaction are promising prognostic markers. In particular, RCC metabolism has recently gained interest during the last years. In RCC and other malignant tumors switch from oxidative phosphorylation to aerobe glycolysis, known as Warburg effect, is observed^5,6^.
- Mitochondria, which are the main cellular energy suppliers, are involved in the Warburg effect. In cancer, they are additionally involved in cell invasion and programmed cell death^7^. Von Hippel-Lindau (VHL) gene inactivation is the most common alteration in sporadic ccRCC^8^. VHL deficiency induces overexpression of hypoxia-inducible factor 1 (HIF-1), which negatively regulates mitochondrial mass and oxygen intake in ccRCC^9^.

A significantly lower amount of mitochondrial DNA and genes coded by mitochondrial DNA have been described in RCC^10,11^. This suggests that mitochondrial processes, including mitophagy, are impaired in this cancer entity. Mitophagy is an autophagic process aimed at the degradation of dysfunctional mitochondria. PINK1-PARK2-mediated signaling is one of the best-described mitophagy pathways^12,13^. Under normal conditions, the PTEN-induced putative kinase 1 (PINK1) is translocated towards the inner mitochondrial membrane, where it is cleaved and subsequently degraded. Mitochondrial depolarization causes PINK1 accumulation on the outer mitochondrial membrane, which induces (cytosolic) PARK2 recruitment^14^. PARK2 is an E3-ubiquitin protein ligase, involved in autophagosome formation and lysosomal degradation^15–17^. Dysregulated mitophagy resulting in the accumulation of damaged mitochondria and increased ROS levels plays an important role in cancer. Still, the exact mechanisms remain unclear^18^. Earlier studies indicated that the downregulation of *PARK2* may serve as a prognostic marker in clear cell renal cell carcinoma^19,20^. Here, we sought to investigate the role of *PINK1* and *PARK2* in pRCC, in particular, its correlation with clinical-pathological parameters and survival.

## Material and methods

### The cancer genome atlas (TCGA) cohort

We extracted clinical (version 28.01.2016) and normalized mRNA expression data (Illumina HiSeq 2000 RNA Sequencing platform, version 2) for pRCC from the TCGA cohort. After screening for consistency, clinical data from 285 patients with available follow-up information and median follow-up duration of 25.6 months (1.0–131.7 mo; overall survival (OS)) were included. During the follow-up 42 patients died.. In 33 cases, normal renal tissue was available for comparative mRNA expression analysis.

### TCGA cohort: statistical analysis

Statistical analysis was performed in the R environment (R Foundation for Statistical Computing; version 3.6.0). For descriptive statistics, Mann–Whitney U-test was used for comparison between groups. Kaplan–Meier estimates, log-rank test, as well as univariate and multivariate Cox proportional hazards analyses were used for survival analysis with overall survival as endpoint. In the univariate Cox analysis all parameters which were significantly associated with overall survival were identified (*p* ≤ 0.05; with only exclusion made for the histological subtype of papillary RCC which showed marginal *p* = 0.051 in TCGA cohort). Next, all significant variables were entered simultaneously into a multivariate model. Using stepwise backward conditional elimination, parameters losing statistical significance were left out. Except for the patient age at operation, all variables were handled as categorial variables. We extended the *p*-value to further observe statistical trends (*p* ≤ 0.1) in the immunohistochemistry cohort, which was restricted by a relatively low number of patients. Median expression levels and optimized cut-offs (sequential analysis of all possible cut-offs using survMisc package in R) were used for categorization of expression levels.

### The tissue microarray (TMA) patient cohort

Tissue samples from 95 patients who underwent partial or radical nephrectomy for pRCC were included. The patients were treated at Charité Berlin University of Medicine (n = 36) or at Erlangen University Hospital (n = 59) between 1995 and 2004. The median age was 62.5 years (range 28–89 years). The median follow-up was 52.2 months (range 0–228 months). Patients were classified according to TNM classification (8th edition). Presence of distant metastases was evaluated at the time of presentation. Clinical and histopathological parameters are displayed in Table 1. TMAs were constructed from formalin-fixed, paraffin-embedded material.

**Table 1:** Histopathological parameters of the patient cohorts.

	TCGA cohort (n = 285)		TMA cohort (n = 95)	
	n	[%]	n	[%]
**Gender**				
Male	210	73.7	76	80.0
Female	75	26.3	17	17.9
Unknown	-	-	2	2.1
**pT**				
pT1	192	67.4	63	66.3
pT2	33	11.6	9	9.5
pT3	60	21.1	18	18.9
pTx	-	-	5	5.3
**pN**				
pN0/cN0	259	90.9	90	94.7
pN1	26	9.1	5	5.3
**M**				
M0	277	97.2	86	90.5
M1	8	2.8	9	9.5
**Grade**				
G1/G2	N/A^§^	N/A	77	81.1
G3/G4	N/A^§^	N/A	18	18.9
**Histological subtype**				
Type 1	76	26.7	N/A*	N/A
Type 2	84	29.5	N/A*	N/A
Unknown	125	43.8	N/A*	N/A

^§^Grading information was not available; *histological subtype classification was not available.

IHC staining of PINK1 and PARK2 and statistical analysis.

All TMAs were stained on a Ventana BenchMark Ultra Autostainer (Roche Diagnostics, Switzerland) for PINK1 with the anti-PINK1 antibody ab23707 (Abcam, UK) at a dilution of 1:30 and anti-PARK2 antibody sc32282 (Santa Cruz, USA) at a dilution of 1:50. Antigen retrieval was performed with antigen retrieval solution CC1 at pH8 (Ventana, Roche Diagnostics Switzerland). Immunohistochemical staining was assessed using a Leica DM 500 microscope (Leica, Germany).

The intensity of the PINK1 and PARK2 expression was assessed on a scale from 0 to 3 (0 = negative, 1 = weak, 2 = moderate, 3 = strong; Fig. 2) and the mean value per case was used for further statistical analysis, if several spots were available on the TMA. Additionally, 28 non-neoplastic renal samples were stained for PINK1 and 26 for PARK2.

The protein expression was dichotomized in low (no staining or 1 +) and high (2 + and 3 +) expression. For correlation analysis with histopathological parameters, we used the Mann–Whitney-U test, a *p*-value ≤ 0.05 was considered significant. Survival analysis was performed with Cox regression analysis and Kaplan–Meier survival analysis including the log-rank test. Statistical analyses were performed using the SPSS software v.25.0 (IBM, USA).

### Ethical approval

The study was approved by the ethic committees of University Erlangen (3755/2008 and 329_16B/2016) and of Medical Faculty Bonn (EK 219/17). All procedures were performed in accordance with ethical standards established in the 1964 Declaration of Helsinki and its later amendments. All patients treated from 2008 gave informed consent. For patients treated before 2008, the ethic committee waived the need for informed individual consent.

### Consent for publication

All authors read and approved the manuscript.

## Results

### Expression of PINK1 and PARK2 was reduced in pRCC

**Figure 1:**
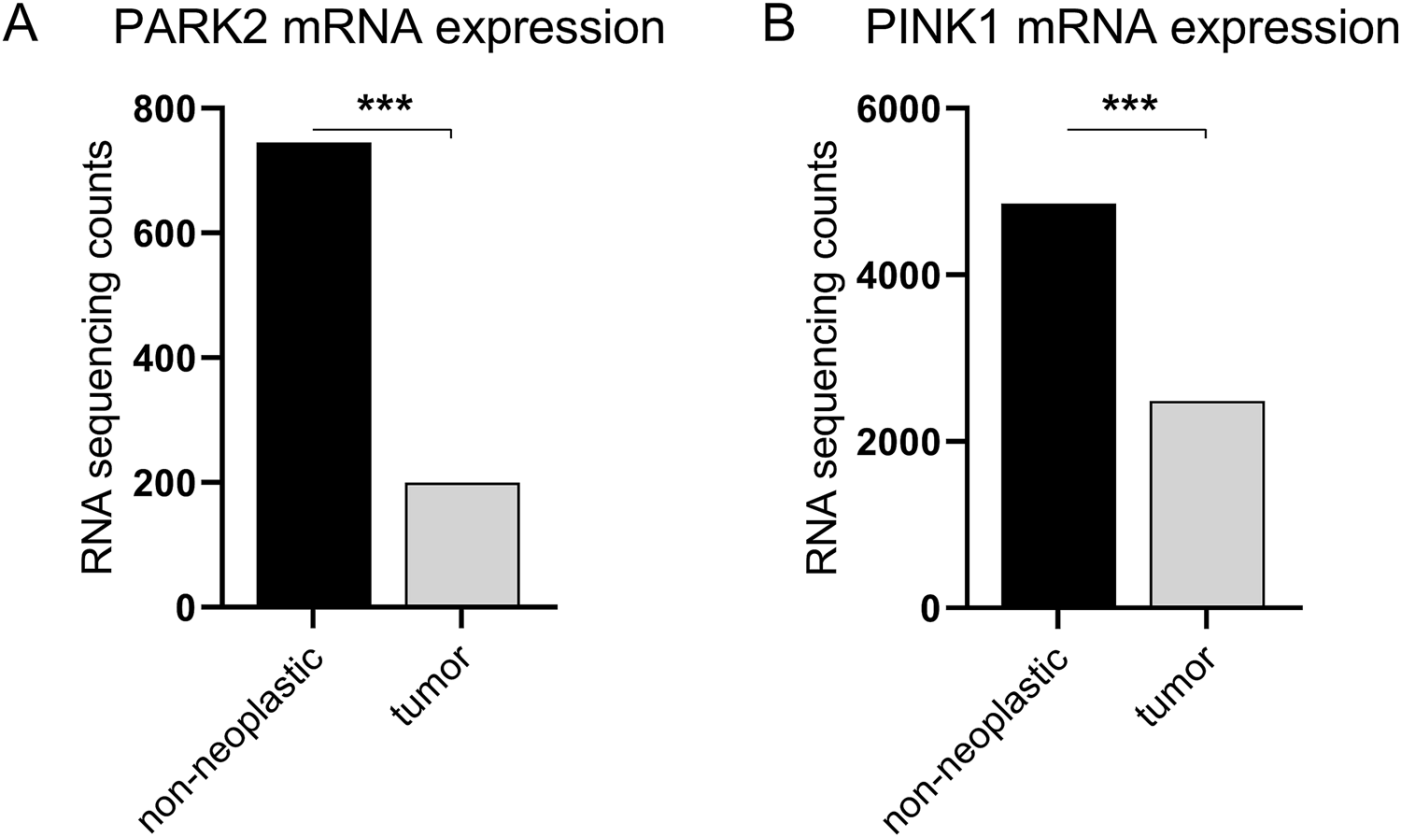
*PARK2* (**A**) and *PINK1 *(**B**) mRNA expression in tumor and non-neoplastic tissue from the TCGA dataset. RNA sequencing counts were plotted. ***p < 0.001.

**Figure 2:**
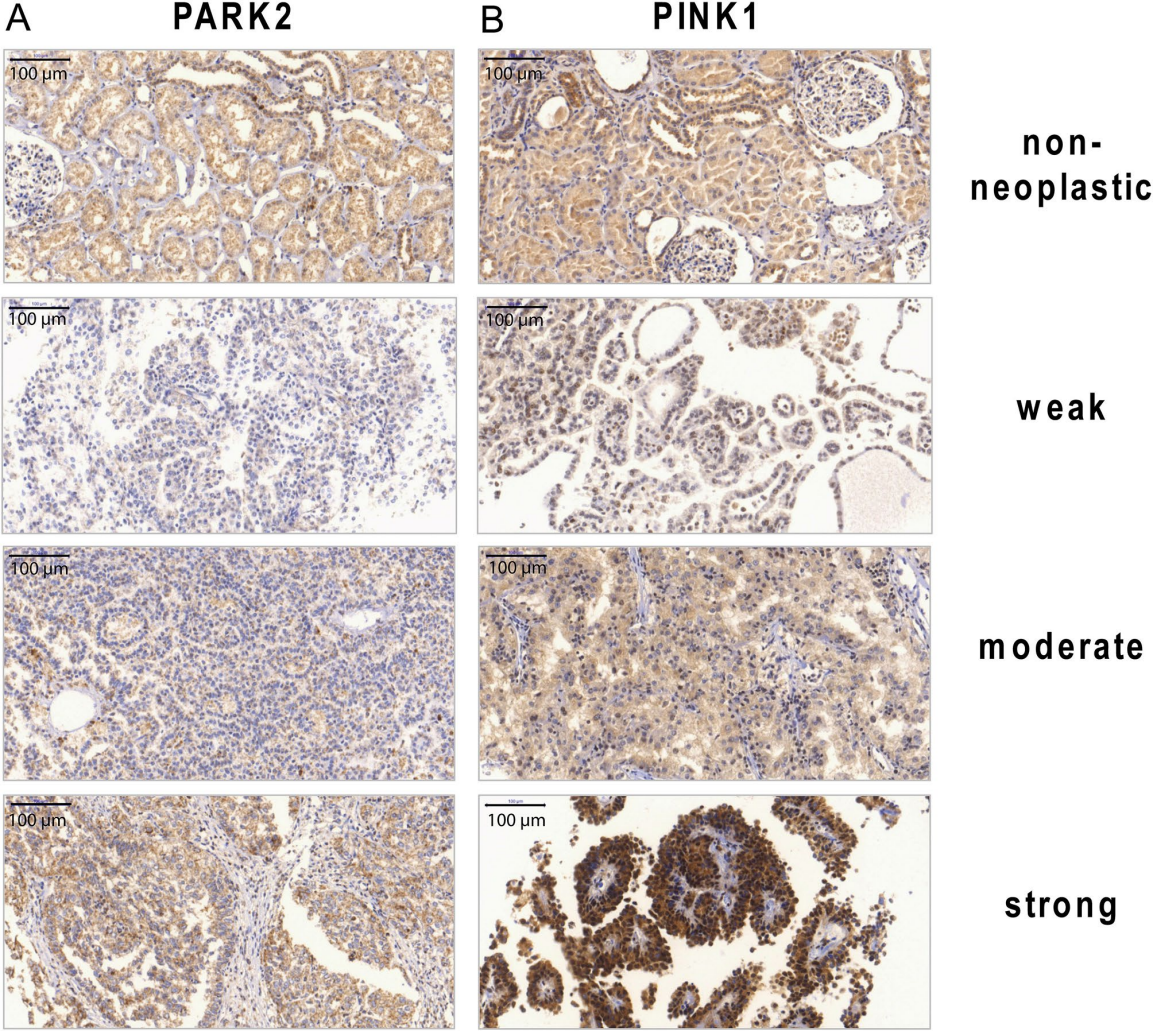
IHC staining of tissue microarrays for protein expression of PINK1 and PARK2. The staining was assessed using a semi-quantitative scale (0 = negative, 1 = weak, 2 = moderate, 3 = strong expression). (**A**) Expression of PINK1 in non-neoplastic and neoplastic tissue. (**B**) Expression of PARK2 in non-neoplastic and neoplastic tissue. Magnification × 200.

The mean mRNA expression of *PARK2* and *PINK1* was significantly lower in tumors compared to non-neoplastic tissue. Tumor expression levels displayed a wide variation (Fig. 1). Protein expression was evaluable in 76 cases for PINK1 and in 63 cases for PARK2. The remaining specimens were not stained sufficiently: in some cases, tumor cells were missing and only fibrotic tissue was transferred to the microarray. These cases were excluded. For PINK1, 14.5% of the samples (11/76) were negative, 40.8% (31/76) were weakly, 27.7% (21/76) moderately and 17.1% (13/76 tumors) strongly stained (Fig. 2B).

**Figure 3:**
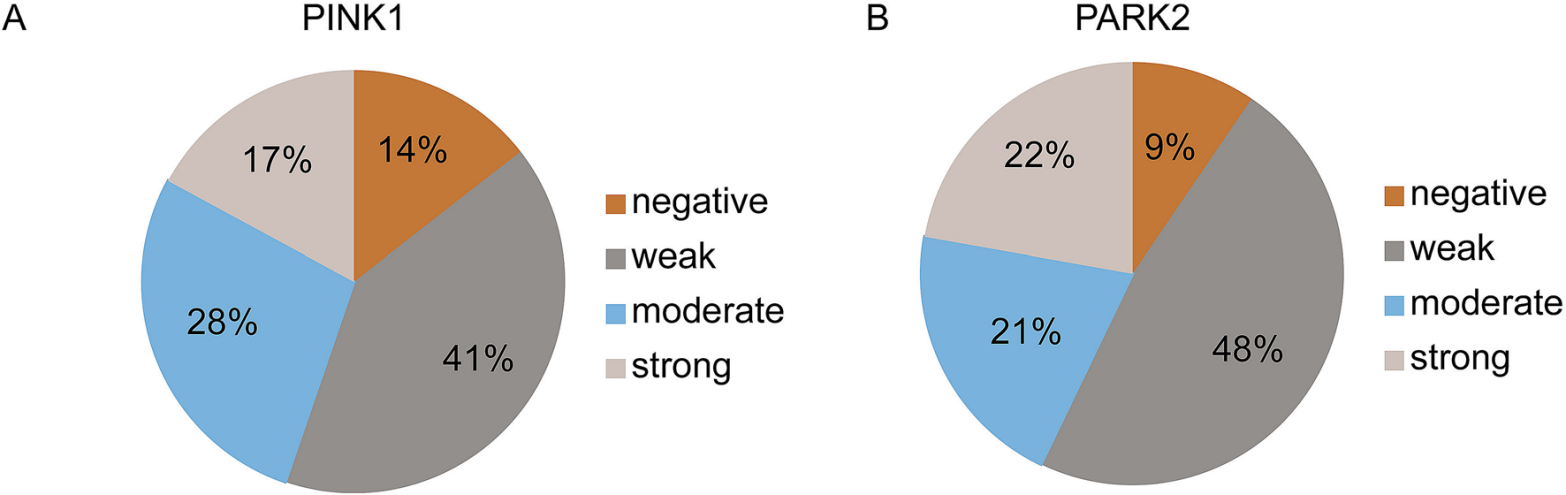
Pie chart diagram of the immunohistochemical staining intensity of PINK1 and PARK2. (**A**) PINK1 was commonly not or only weakly expressed in pRCC samples. (**B**) The PARK2 expression was weak or negative in the majority pRCC patients.

A negative PARK2 staining was observed in 9.5% (6/63), weak intensity in 47.6% (30/63), moderate staining in 20.6% (13/63) and a strong expression in 22.2% (14/63) (Fig. 2A). In conclusion, more than half of all pRCC specimens displayed an absent or weak PINK1 (53.3%) and PARK2 (57.1%) protein expression (Fig. 3A,B, respectively).

2 (7.1%) non-neoplastic renal tissue samples were negative for PINK1, 21 (75.0%) were weakly positive and 5 cases displayed a moderate positivity (17.9%). For PARK2, 3 (11.5%) samples were negatively, 10 (38.5%) weakly, 10 (38.5%) moderately and 3 (11.5%) strongly stained.

Compared to non-neoplastic kidney tissue, the pRCC specimens displayed lower PINK1 levels. However, only a statistical trend was observed (Mann–Whitney test, *p* = 0.077). No difference in PARK2 expression between pRCC tissue and non-neoplastic renal tissue was noted.

### PINK1 expression is negatively correlated with adverse pathology and occurrence of metastases in pRCC

*PINK1* and *PARK2* mRNA expression in the tumor tissue was weakly correlated (Pearson r = 0.19, *p* = 0.002). *PINK1* mRNA expression was negatively correlated with pT-stage (Pearson r = -0.19, *p* = 0.001), pN-status (Pearson r = -0.17, *p* = 0.004) and presence of distant metastases at the time of presentation (Pearson r = -0.13, *p* = 0.024). In addition, it was positively correlated with histological type (types 1 or 2) with higher expression in type 2 carcinomas (Pearson r = 0.19, *p* = 0.016). *PARK2* mRNA expression was not correlated with any pathological parameters.

PINK1 and PARK2 protein expression levels, assessed by staining intensity, were significantly correlated (Spearman’s rho = 0.29; *p* = 0.040). Patients with metastasized pRCC had significantly lower levels of PINK1 protein expression compared to patients without metastases at presentation (Mann–Whitney U-test, *p* = 0.030, z = (-2.171), r = 0.249). No significant difference in the PINK1 protein expression with regard to the pN-status or histological grade was noticed. No significant correlation between pathological parameters and PARK2 protein expression was observed.

### Downregulation of PINK1 and PARK2 mRNA is an independent prognostic factor for poor survival

**Figure 4:**
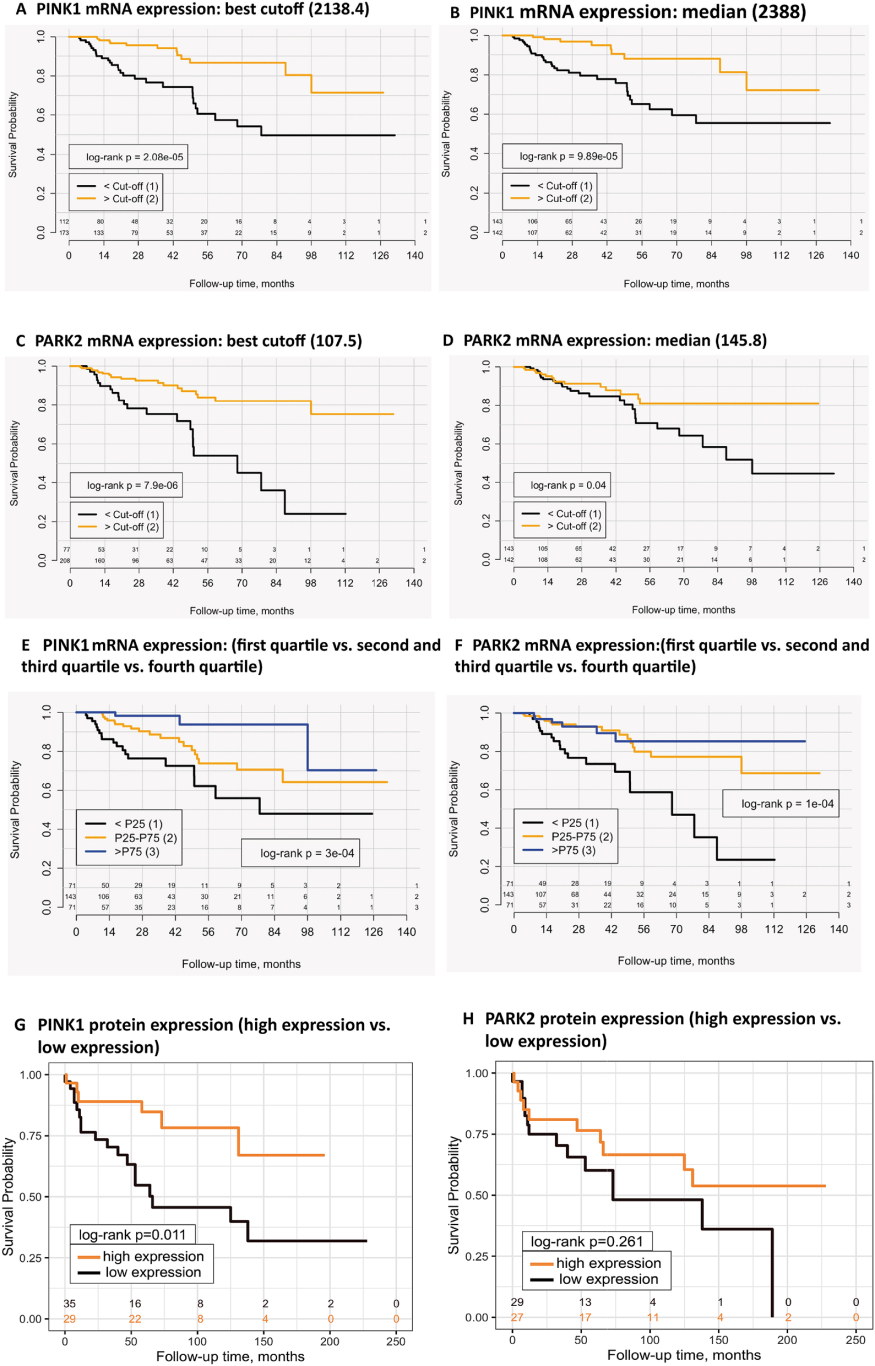
Reduced mRNA and protein expression of PINK1 and PARK2 are associated with shorter overall survival. The mRNA expression levels of *PINK1* were dichotomized by the best cutoff (**A**) and the median (**B**); A lower *PINK1* expression was highly significantly associated with an adverse prognosis. The same adverse effect was observed for lower mRNA expression levels of *PARK2* after dichotomization (**C**, **D**). Also when comparing the first quartile to the fourth quartile and combining the second and third quartiles as an intermediate group, the mRNA expression levels of *PINK1* (**E**) and *PARK2* (**F**) are significantly associated with prognosis. The protein expression was dichotomized in high (moderate or strong expression) and low (negative or weak expression). Patients with low protein expression of PINK1 had a significantly lower overall survival (in months) compared to patients with high protein staining (**G**). No significant association with survival was noticed for PARK2, protein expression (**H**); Kaplan Meier plots, including a log-rank test with *p* ≤ 0.05 being significant.

Patients with an increased *PINK1* and *PARK2* expression (using cutoff dichotomization) had a significantly longer overall survival compared to patients with downregulated *PINK1* and *PARK2* levels in Kaplan Meier and univariate Cox-analysis (Fig. 4A–F). Univariate Cox analysis results revealed that expression levels of PINK1 and PARK2 and the clinico-pathological variables were statistically significant associated with survival (*p* ≤ 0.05); The histological subtype of pRCC was marginally not significantly associated (*p* = 0.051) (Table 2). Sex of the patient and age at operation were not significantly associated with overall survival in the cohort. Comorbidities, overall performance and histological grading were not available for analysis.

**Table 2:** Univariate and multivariate Cox analysis for *PINK1* and *PARK2* mRNA expression in TCGA cohort (overall survival as endpoint).

Parameter	Univariate Cox analysis			Multivariate Cox analysis		
	HR	95%CI	*p*-value	HR	95%CI	*p*-value
PINK1*						
> Cut-off	1.0	–	–	1.0	–	–
< Cut-off	3.8	2.0–6.8	3.0e−05	3.6	1.8–7.2	0.0003
PARK2**						
> Cut-off	1.0	–	–	1.0	–	–
< Cut-off	3.8	2.0–7.2	7.4e−05	2.4	1.3–4.4	0.008
**pT-stage**						
pT1	1.0	–	–	1.0	–	–
pT2	3.1	1.3–7.5	0.012	2.3	0.9–5.9	0.068
pT3	7.0	3.5–14.0	3.9e−08	3.0	1.3–7.3	0.012
**pN-status**						
pN0/cN0	1.0	–	–	1.0	–	–
pN1	9.6	5.1–17.7	8.3e−13	4.1	1.8–9.6	0.001
**Histological subtype**						
Type 1	1.0	–	–			
Type 2	2.7	1.0–7.8	0.051			
**M-status**						
M0	1.0	–	–	1.0	–	–
M1	101.3	31.9–321.7	4.7e−15	14.9	4.1–53.8	3.7e−05

Comments: *—optimized cut-off was used for dichotomization (number of transcripts = 2138).

**- optimized cut-off was used for dichotomization (number of transcripts = 108).

In the multivariate Cox-analysis with pT-stage, pN-status and M-status as well as increased *PARK2* and *PINK1* mRNA expression retained independent prognostic significance with regard to overall survival, while histological subtype was removed from the model as non-significant, forming the final version of the model (Table 2).

### PINK1 and PARK2 protein expression and their association with OS

For survival analysis, tumor samples were dichotomized into tumors with absent or low PINK1 expression (low) and tumors with moderate to high PINK1 expression (high). Patients with low PINK1 protein expression were at risk of shorter OS than patients with high PINK1 expression (HR = 3.1, 95%, CI 1.2 – 8.0, *P* = 0.016, Fig. 4G). In our small cohort, age, sex and ISUP grade did not show any meaningful association with overall survival in univariate analysis (all *p* > 0.05) and, therefore, were not included into multivariate model. Also, PARK2 protein expression was not statistically significant associated with overall survival (Fig. 4H). In the multivariate Cox regression model, we relaxed the *p*-value threshold to 0.1 to be able to see statistical trends (due to relatively small number of patients in the cohort). The model included PINK1 protein expression, pT-, pN-stages and M-Status, whereby M-status was lacking an independent prognostic value and was excluded in backward elimination, leaving the final version of the model presented in Table 3. Low PINK1 protein expression had an independent prognostic value with HR 3.61 (95%CI 1.2–11.0, *p* = 0.024) compared to high protein expression.

**Table 3:** Univariate and multivariate Cox analysis for PINK1 and PARK2 protein expression (overall survival as endpoint).

Parameter	Univariate Cox analysis			Multivariate Cox analysis		
	HR	95%CI	*p*-value	HR	95%CI	*p*-value
**PINK1**						
High	1.0	–	–	1.0	–	–
Low	3.1	1.2–8.0	0.016	3.61	1.2–11.0	0.024
**PARK2**						
High	1.0	–	–			
Low	1.6	0.7–3.7	0.263			
**pT-stage**						
pT1	1.0	–	–	1.0	–	–
pT2 or pT3	2.80	1.3–6.0	0.008	2.3	1.0–5.5	0.059
**pN-status**						
pN0/cN0	1.0	–	–	1.0	–	–
pN1	5.2	1.9–14.0	0.001	2.7	0.8–8.6	0.09
**M-status**						
M0	1.0	–	–			
M1	7.34	3.05–17.62	9.7e−06			

## Discussion

pRCC is the second most common subtype of renal malignant tumors. Nonetheless, little is known about its molecular-pathological characteristics. Prognostic markers for clinical patient care and disease management are scarce. Hence, efforts have been made to investigate potential prognosticators in pRCC.

Gao et al. described a mRNA signature consisting of five genes (*CCNB2*, *IGF2BP3*, *KIF18A*, *PTTG1*, and *BUB1*) associated withOS^21^. Another study identified *KPNA2* mRNA levels as an prognostic factor for worsened OS in pRCC^22^. However, tumor stage and grading are still the most important factors predicting survival in pRCC^23–25^. This clearly demonstrates the urgent need for further research.

In this study, we investigated the prognostic value of two mitophagy-associated genes in pRCC, namely *PINK1* and *PARK2.* . We observed a significantly lower expression of *PINK1* and *PARK2* in pRCC compared to non-neoplastic tissue. Additionally, a significant downregulation of PINK1 and PARK2 protein expression was found.

In a previous study, we described similar results for *PARK2* in ccRCC. In fact, lower PARK2 mRNA levels were associated with tumor aggressiveness and adverse prognosis, while the protein expression did not correlate with pathological parameters and overall survival^20^. In our cohort of pRCC patients, we did not find any correlations between PARK2 and TNM stage or grading. However, low mRNA levels of PARK2 correlated with shorter OS.

*A PARK2* downregulation has been described in many solid tumors, such as osteosarcoma, colorectal cancer, breast cancer, pancreatic cancer and lung cancer^26–30^. In lung cancer and osteosarcoma, downregulation of PARK2 was associated with poor prognosis and higher TNM stage^26,30^. In our study, we noticed a significantly lower *PINK1* expression in tumor compared to non-neoplastic tissue.

Data regarding *PINK1* in pRCC are scarce. Reduced mitochondrial function due to diminished mitochondrial DNA and RNA was found in pRCC^10,11^.

Our results support the theory that reduced mitophagy occurs in this rare renal cancer subtype. However, the consequences of decreased mitophagy in pRCC remain unclear.

A low *PINK1* mRNA expression was significantly related to higher tumor stage and positive nodal status. . Moreover, a low mRNA and protein expression of PINK1 was an independent prognostic factor for OS in our cohort. In addition, it was associated with the presence of distant metastases at the time of presentation.

The consequences of *PARK2* and *PINK1* dysregulation in cancer are not fully elucidated. One of the key functions of PINK1 and PARK2 is the clearance of damaged mitochondria through the PINK1/PARK2 axis. PARK2 mutations affect the PINK1/PARK2 mitophagy axis in lung cancer due to slower clearance of damaged mitochondria^31^. Morever, dysregulation of the PINK1/PARK2 axis accelerates KRAS-mediated carcinogenesis in pancreatic cancer. This dysregulation leads to mitochondrial iron accumulation and inflammasome activation in pancreatic tumor cells^29^. The PINK1/PARK2 pathway and its association with poor prognosis needs further investigations. Iron or calcium accumulation and inflammasome activation due to impaired mitochondrial clearance might be explained by a switch to glycolysis, an activation of hypoxia inducible factors and various growth factors. RCC metabolism, especially anaerobe glycolysis in ccRCC, has been successfully targeted on a molecular level in vitro^32^. However, further research should focus on a possible therapeutic targeting of mitophagy in RCC. Mitochondrial turnover and mitochondrial degradation might be crucial in pRCC metabolism and cell biology, therefore, these mechanisms in pRCC need to be further investigated.

## Conclusions

To our knowledge, this is the first report providing a detailed analysis of mitophagy-related *PINK1* and *PARK2* expression (mRNA and protein) in pRCC. We found a significant association between *PINK1* and *PARK2* downregulation and OS. Therefore, it is reasonable to assume that *PINK1* and *PARK2* are potential prognosticators in this rare cancer subtype. In the future, PINK1 protein expression could be included in routine diagnostic protocols and guide disease management. However, larger patient cohorts are necessary to validate these genes as prognostic markers in a clinical setting.

## Data Availability

mRNA expression data were obtained from TCGA. Protein expression data generated and analyzed in this study are included in the article.

## Abbreviations

RCC: Renal cell carcinoma
pRCC: Papillary renal cancer
PARK2: Parkin 2
PINK1: PTEN-induced kinase 1
TMA: Tissue microarrays
OS: Overall survival
